# Economic and health impacts of introducing *Helicobacter pylori* eradication strategy into national gastric cancer policy in Japan: A cost‐effectiveness analysis

**DOI:** 10.1111/hel.12837

**Published:** 2021-07-18

**Authors:** Akiko Kowada, Masahiro Asaka

**Affiliations:** ^1^ Department of Occupational Health Kitasato University Graduate School of Medical Sciences Kanagawa Japan; ^2^ Advanced Research Promotion Center Health Sciences University of Hokkaido Ishikari gun Japan

**Keywords:** cancer prevention, cost‐effectiveness, gastric cancer, health economics, *Helicobacter pylori* eradication

## Abstract

**Background:**

*Helicobacter pylori* (*H. pylori*) eradication reduces gastric cancer risk. Since 2013, a population‐wide *H. pylori* eradication strategy for patients with chronic gastritis has begun to prevent gastric cancer in Japan. The aim of this study was to evaluate the economic and health effects of *H. pylori* eradication strategy in national gastric cancer prevention program.

**Materials and Methods:**

We developed a cohort state‐transition model for *H. pylori* eradication and no eradication over a lifetime horizon from a healthcare payer perspective, and performed one‐way and probabilistic sensitivity analyses. We targeted a hypothetical cohort of *H. pylori*‐positive patients aged 20, 30, 40, 50, 60, 70, and 80. The main outcomes were costs, quality‐adjusted life‐years (QALYs), life expectancy life‐years (LYs), incremental cost‐effectiveness ratios, gastric cancer cases, and deaths from gastric cancer.

**Results:**

*H. pylori* eradication was more effective and cost‐saving for all age groups than no eradication. Sensitivity analyses showed strong robustness of the results. From 2013‐2019 for 8.50 million patients, *H. pylori* eradication saved US$3.75 billion, increased 11.11 million QALYs and 0.45 million LYs, and prevented 284,188 cases and 65,060 deaths. For 35.59 million patients without eradication, *H. pylori* eradication has the potential to save US$14.82 billion, increase 43.10 million QALYs and 1.66 million LYs, and prevent 1,084,532 cases and 250,256 deaths.

**Conclusions:**

National policy using population‐wide *H. pylori* eradication to prevent gastric cancer has significant cost savings and health impacts for young‐, middle‐, and old‐aged individuals in Japan. The findings strongly support the promotion of *H. pylori* eradication strategy for all age groups in high‐incidence countries.

## 1 INTRODUCTION

**FIGURE 1:**
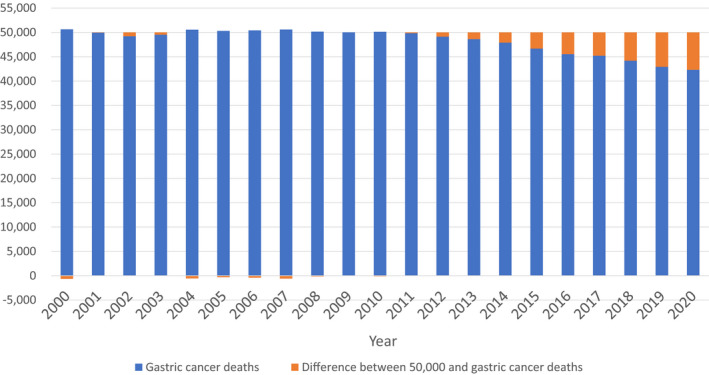
Changes in gastric cancer deaths in Japan from 2000 to 2020

More than half of the world's population is infected with *Helicobacter pylori* (*H. pylori*).^1^ *H. pylori* infection causes chronic atrophic gastritis, a common stage of progression to gastric cancer, and is responsible for 98% of the causes of gastric cancer in Japan.^2^, ^3^, ^4^, ^5^ Japan has the third highest age‐standardized rate for gastric cancer in the world.^6^ The incidence of gastric cancer in Japan is almost 10 times higher than that observed in the United States. The Taipei global consensus guidelines for screening and eradication of *H. pylori* for gastric cancer prevention recommend that mass screening and eradication of *H. pylori* should be considered in populations at higher risk of gastric cancer and that eradication therapy should be offered to all individuals infected with *H. pylori*.^7^ In the guidelines for the management of *H. pylori* infection by the Japanese Society for Helicobacter Research, *H. pylori* eradication treatment is recommended to prevent gastric cancer for patients with *H. pylori* infection.^8^ The Ministry of Health, Labour and Welfare approved expansion of National Health Insurance coverage for *H. pylori* eradication treatment in patients with chronic gastritis from February 2013.^9^ During 2013‐2019, 8.50 million *H. pylori*‐positive patients received eradication treatment.^10^, ^11^ The number of deaths from gastric cancer is gradually declining, with 42,931 deaths in 2019 and 42,318 deaths in 2020 (Figure 1).^12^, ^13^

In this study, we aimed to evaluate the economic and health effects of *H. pylori* eradication strategy in national gastric cancer prevention program in Japan.

## 2 MATERIALS AND METHODS

### 2.1 Study design and model structure

**FIGURE 2:**
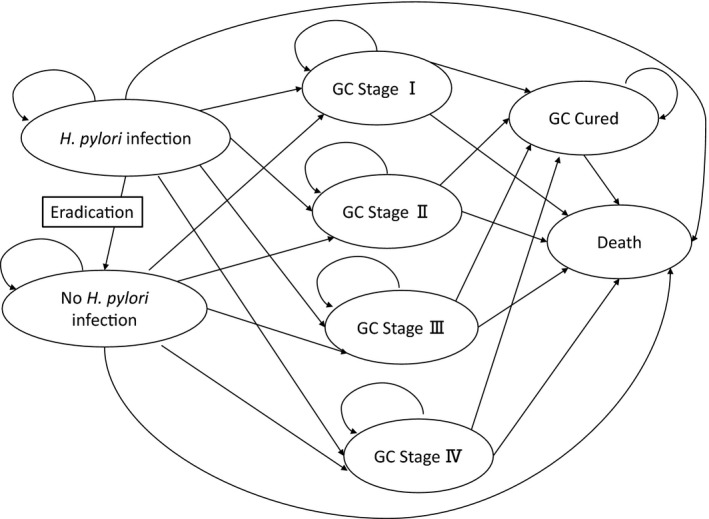
Schematic depiction of the Markov cycle tree in the cohort state‐transition model. We show that health states in the model as ovals. In a yearly model cycle, transitions can occur between the health states and other health states, represented by the arrows. *H*. *pylori* = *Helicobacter pylori*; GC = gastric cancer

We constructed a cohort state‐transition model with a Markov cycle tree for two strategies: *H*. *pylori* eradication strategy and no eradication strategy, using a healthcare payer perspective and a lifetime horizon. A cycle length of one year was chosen. The half‐cycle correction was applied. In the model, decision branches leaded directly to one Markov node per intervention strategy and the first events were modeled within the Markov cycle tree (Figure 2). We used TreeAge Pro (TreeAge Software Inc., Williamstown, Massachusetts) for the Decision‐analytical calculations. As this was a modeling study with all inputs and parameters derived from the published literature and Japanese statistics, ethics approval was not required.

#### 2.1.1 H. pylori eradication strategy

The *H. pylori*‐positive patient receives first‐line eradication treatment (proton‐pump inhibitor, clarithromycin, and amoxicillin). The patient who fails first‐line eradication treatment receives second‐line eradication treatment (proton‐pump inhibitor, metronidazole, and amoxicillin). We consider the eradication and compliance rates of first‐line and second‐line eradication treatments in the model. After successful *H. pylori* eradication, *H. pylori‐*positive changes to *H. pylori‐*negative. When the patient fails both treatments, *H. pylori*‐positive remains until death. We calculate the costs of *H. pylori* test, endoscopy, and two urea breath tests when the patient receives *H. pylori* eradication treatment. When the patient has gastric cancer, the patient receives the standard treatment of gastric cancer followed by the Japanese gastric cancer treatment guidelines: endoscopic mucosal resection (EMR), endoscopic submucosal dissection treatment (ESD), surgery, chemotherapy, and radiotherapy with palliative care according to cancer stages, stage I‐IV.^14^ The model includes the relative risk of developing gastric cancer after successful eradication, stage‐specific 5‐year survival rates, and mortality due to other causes (Table 1).^12^, ^15^ The patient aged 50 and over receives endoscopic screening every year from the year after eradication.

**TABLE 1:** Baseline estimates for selected variables

Variable	Baseline value		Sensitivity analysis range	Reference
Incidence of gastric cancer in *H. pylori*‐positive patients				
20y 30y 40y 50y 60y 70y 80y	0.000771 0.001167 0.001881 0.002803 0.005122 0.007949 0.009117		0.0001‐0.01	2,3,4,5,12,16
Incidence of gastric cancer in *H. pylori*‐positive patients after successful eradication treatment				
20y 30y 40y 50y 60y 70y 80y	0.000509 0.00077 0.00124 0.00185 0.00338 0.00524 0.00602		0.0001‐0.01	2,3,4,5,12,15,16
Prevalence of *H. pylori* infection (%)				
20y 30y 40y 50y 60y 70y 80y	6.1 14.7 23.7 33.7 47.7 58.6 63.6		1‐80	16
Stage‐specific 5‐year gastric cancer survival rate (%)				
Stage I Stage II Stage III Stage IV	96.0 69.2 41.9 6.3		90‐99 50‐80 30‐50 0‐20	12
Number of *H. pylori*‐positive patients with eradication from 2013 to 2019				
20‐29y 30‐39y 40‐49y 50‐59y 60‐69y 70‐79y 80‐89y	123,986 422,965 1,083,631 1,664,732 2,860,031 1,903,756 440,503		N/A	10, 11, expert opinion
Number of *H. pylori*‐positive patients who do not receive *H. pylori* eradication treatment				
20‐29y 30‐39y 40‐49y 50‐59y 60‐69y 70‐79y 80‐89y	773,480 2,034,480 4,256,520 5,634,640 7,331,490 9,622,120 5,933,880		N/A	16, national census
Eradication rate of first‐line eradication treatment with proton‐pump inhibitor, amoxicillin, and clarithromycin for 1 week				
	0.798		0.6‐1.0	19
Eradication rate of second‐line eradication treatment with proton‐pump inhibitor, amoxicillin, and metronidazole for 1 week				
	0.837		0.6‐1.0	19
Relative risk of gastric cancer development after successful eradication treatment				
		0.66	0.46‐0.95	15
Compliance rate for first‐line eradication treatment				
		0.848	0.6‐1.0	19
Compliance rate for second‐line eradication treatment				
		0.678	0.6‐1.0	19
Responsibility rate of *H. pylori* infection for gastric cancer development				
		0.98	N/A	2,3,4,5
Sensitivity of endoscopy		0.954	0.842‐0.994	20
Specificity of endoscopy		0.888	0.883‐0.892	20
Proportion of gastric cancer stage at initial screening (%)				
Stage I Stage II Stage III Stage IV		62.5 11.0 7.5 19.0	30‐80 5‐20 2‐15 10‐50	12
Costs, US$ (US$ = ¥ 100.64)				
*H. pylori* test		7.9	4.0‐15.9	17
Urea breath test		7.0	3.5‐14.0	
First‐line *H. pylori* eradication treatment		42.8	21.4‐85.6	
Second‐line *H. pylori* eradication treatment		38.9	19.5‐77.8	
Endoscopy	113.3		56.6‐226.6	
Treatment of gastric cancer			
Stage I Stage II Stage III Stage IV	3675 15,898 24,841 29,809		1838‐7350 7949‐31,796 12,421‐49,682 14,905‐59,618
Utilities				
No *H. pylori* infection		1	N/A	
*H. pylori* infection		0.9	0.8‐0.95	25,26
Gastric cancer				
Stage I	0.82		0.7‐0.9	
Stage II	0.79		0.7‐0.9	
Stage III	0.68		0.6‐0.8	
Stage IV	0.5		0.4‐0.6	
Cured	0.95		0.92‐0.97	
Death	0		N/A

Abbrevations *H. pylori* = *Helicobacter pylori*; N/A = not applicable

#### 2.1.2 No eradication strategy

The latest version of Japanese guidelines for effective secondary prevention of gastric cancer recommends upper gastrointestinal series and endoscopy in adults 50 years of age and older. In the model, the *H. pylori*‐positive patient does not receive *H. pylori* eradication treatment, and the patient aged 50 and over receives annual endoscopic screening annually. When the patient has gastric cancer, the patient receives the standard treatment of gastric cancer.

### 2.2 Target population

We targeted a hypothetical cohort of Japanese *H. pylori*‐positive chronic gastritis patients who had the initial endoscopic diagnosis needing *H. pylori* eradication at the age of 20, 30, 40, 50, 60, 70, and 80. Children and adolescents (age <20 y) were not included in the model.

### 2.3 Epidemiologic parameters and clinical probabilities

Epidemiologic parameters and clinical probabilities were collected using MEDLINE from 2000 to June 2, 2021, national census, and Japanese cancer statistics (Table 1).^2^, ^3^, ^10^, ^11^, ^12^, ^15^, ^16^, ^17^, ^18^, ^19^, ^20^ We estimated annual age‐specific numbers of *H. pylori*‐positive patients with eradication treatment from the literature^10^, ^11^ and expert opinion (Table1, Figure S1A). The numbers of *H. pylori*‐positive patients with and without eradication were estimated from the literature^16^ and national census (Table1, Figure S1B). Relative risk of gastric cancer development after successful eradication^15^, and eradication and compliance rates of first‐ and second‐line eradication treatments^19^ were obtained from the literature. Age‐specific gastric cancer incidence and stage‐specific 5‐year survival rate were obtained from Japanese cancer statistics.^10^ The responsibility rate of *H. pylori* infection for gastric cancer development was assumed to be 98%.^2^, ^3^, ^4^, ^5^ The incidence of gastric cancer in *H. pylori*‐positive patients was estimated using the responsibility rate of *H. pylori* infection for gastric cancer development and the prevalence of *H. pylori* infection.^2^, ^3^, ^4^, ^5^, ^12^, ^16^ The incidence of gastric cancer in *H. pylori*‐positive patients after successful eradication treatment was estimated using the relative risk of gastric cancer development after successful eradication treatment.^2^, ^3^, ^4^, ^5^, ^12^, ^15^, ^16^ The sensitivity and specificity of endoscopy were obtained from the literature.^20^

### 2.4 Costs

Costs were calculated based on the costs from the Japanese national fee schedule^17^ and were adjusted to 2019 Japanese yen, using the medical care component of the Japanese consumer price index and were converted to US dollars, using the Organisation for Economic Co‐operation and Development (OECD) purchasing power parity rate in 2019 (US$1 = ¥100.64) (Table 1).^21^ All costs were discounted by 3%.^22^, ^23^ Incremental cost‐effectiveness ratios (ICERs) were calculated and compared to two willingness‐to‐pay levels of US$50,000 per quality‐adjusted life‐year (QALY) gained and US$100,000 per QALY gained.^24^ Age‐specific and total cumulative lifetime cost savings of *H. pylori* eradication strategy compared with no eradication strategy were calculated.

### 2.5 Health utilities, effectiveness, and health outcomes

Health status was included to represent possible eight clinical states: (i) No *H. pylori* infection, (ii) *H. pylori* infection, (iii) gastric cancer on stage I; (iv) gastric cancer on stage II; (v) gastric cancer on stage III; (vi) gastric cancer on stage IV; (vii) cured gastric cancer; and (viii) death (Figure 2). Health state utilities were obtained from the literature and were calculated using utility weights (Table 1).^25^, ^26^ The annual discounting of the utilities in this analysis was set at a rate of 3%.^22^, ^23^

The health outcomes were QALYs, life expectancy life‐years (LYs), gastric cancer cases, and deaths from gastric cancer. Age‐specific and total cumulative lifetime health outcomes of *H. pylori* eradication strategy compared with no eradication strategy were calculated and evaluated.

### 2.6 Sensitivity analyses

We performed a one‐way sensitivity analysis to determine which strategy was more cost‐effective when we tested a single variable over a wide range of possible values while holding all other variables constant, and performed a probabilistic sensitivity analysis using a second‐order Monte‐Carlo simulation for 10,000 trials to assess the impact of the uncertainty in the model on the base‐case estimates. The uncertainty had a beta distribution in clinical probabilities and accuracies, and a log‐normal distribution in costs.

## 3 RESULTS

### 3.1 Base‐case analysis

*H. pylori* eradication strategy was less costly and yielded greater benefits than no eradication strategy for all age groups (Table 2). No eradication strategy was dominated for all age groups. The patients aged 40 had the highest per capita cost‐savings. Per capita gains of QALYs in younger patients were higher than in older patients (Table 2). From 2013 to 2019, the patients aged 60 had the highest cost savings and health outcomes (Table S1).

### 3.2 One‐way sensitivity analysis and probabilistic sensitivity analysis

**FIGURE 3:**
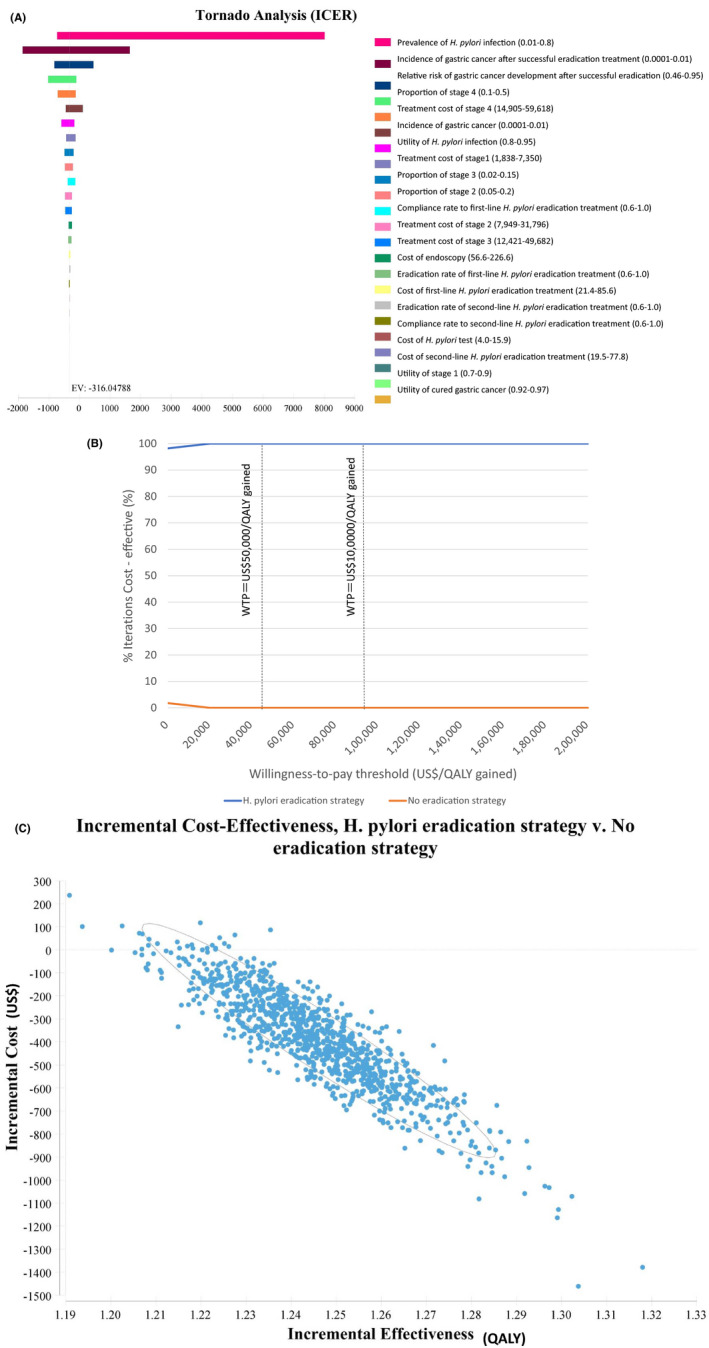
One‐way sensitivity analysis and probabilistic sensitivity analysis in 60‐year‐old *H. pylori*‐positive patients. A, The incremental cost‐effectiveness ratio (ICER) tornado diagram for *H*. *pylori* eradication strategy versus no eradication strategy. The cost‐effectiveness of *H. pylori* eradication strategy was not sensitive to changes in any variables. B, Cost‐effectiveness acceptability curve for *H. pylori* eradication strategy versus no eradication strategy. The probabilistic sensitivity analysis analyzed 10,000 simulations of the model in which input parameters were randomly varied across pre‐specified statistical distributions. The x‐axis represents the willingness‐to‐pay threshold. The acceptability curve showed that *H. pylori* eradication strategy was cost‐effective 100% of the time at two willingness‐to‐pay thresholds of US$50,000 per QALY gained and US$100,000 per QALY gained. C, Incremental cost‐effectiveness scatterplots with 95% confidence ellipses for *H. pylori* eradication strategy versus no eradication strategy. Each dot represents a single simulation for a total of 10,000 simulations. Incremental cost‐effectiveness scatterplots showed that *H. pylori* eradication strategy dominated no‐eradication strategy in 9811 trials, and that *H. pylori* eradication strategy was more cost‐effective than no‐eradication strategy in 189 trials. EV = expected value; *H. pylori* = *Helicobacter pylori*; ICER = incremental cost‐effectivenessratio; QALY = quality‐adjusted life‐year; WTP = willingness‐to‐pay threshold

Incremental cost‐effectiveness ratio tornado diagram of *H. pylori* eradication strategy versus no eradication strategy showed that cost‐effectiveness was not sensitive to any variables in all age groups (Figure 3A, Figure S2).

In probabilistic sensitivity analysis using Monte‐Carlo simulation for 10,000 trials, the acceptability curve showed that *H. pylori* eradication strategy was cost‐effective 100% of the time at two willingness‐to‐pay thresholds of US$50,000 per QALY gained and US$100,000 per QALY gained in all age groups (Figure 3B, Figure S3). Incremental cost‐effectiveness scatterplots showed that *H. pylori* eradication strategy dominated no‐eradication strategy in more than 9800 trials in all age groups (Figure 3C, Figure S4). The results showed strong robustness of *H. pylori* eradication strategy in all age groups.

**TABLE 2:** Results of the base‐case analysis

Age group (y)	Strategy	Cost (US$)	Incremental cost (US$)	Effectiveness (QALYs)	Incremental effectiveness (QALYs)	ICER (US$/ QALY gained)	Life expectancy life‐years (LYs)	Incremental LYs	ICER (US$/LY gained)	Gastric cancer cases (%)	Deaths from gastric cancer (%)
20	*H. pylori* eradication	2473.18	‐	27.0248	‐	‐	27.7464	‐	‐	15.04	3.36
	No eradication	3024.99	551.80	24.9155	‐2.1094	dominated	27.6869	‐0.0594	dominated	19.84	4.43
30	*H. pylori* eradication	2914.97	‐	25.1541	‐	‐	25.8425	‐	‐	14.48	3.25
	No eradication	3614.48	699.50	23.1919	‐1.9623	dominated	25.7768	‐0.0657	dominated	19.12	4.28
40	*H. pylori* eradication	3337.46	‐	22.7218	‐	‐	23.3630	‐	‐	13.60	3.06
	No eradication	4182.64	845.18	20.9509	‐1.7709	dominated	23.2940	‐0.0689	dominated	18.01	4.05
50	*H. pylori* eradication	5666.60	‐	19.6884	‐	‐	20.2624	‐	‐	11.80	2.68
	No eradication	6027.24	360.65	18.1570	‐1.5314	dominated	20.1979	‐0.0644	dominated	15.70	3.56
60	*H. pylori* eradication	5275.75	‐	16.0727	‐	‐	16.5615	‐	‐	9.81	2.26
	No eradication	5669.62	393.87	14.8265	‐1.2462	dominated	16.5066	‐0.0549	dominated	13.14	3.03
70	*H. pylori* eradication	4208.04	‐	11.8844	‐	‐	12.2603	‐	‐	6.63	1.59
	No eradication	4551.33	343.29	10.9703	‐0.9141	dominated	12.2253	‐0.0350	dominated	9.01	2.16
80	*H. pylori* eradication	2651.09	‐	7.5743	‐	‐	7.8248	‐	‐	3.14	0.83
	No eradication	2845.55	194.46	7.0044	‐0.5699	dominated	7.8105	‐0.0143	dominated	4.36	1.14

Abbrevations *H. pylori* = *Helicobacter pylori*; QALY = quality‐adjusted life‐year; LY = life expectancy life‐years; ICER = incremental cost‐effectiveness ratio; dominated = less effective and more costly than others;

### 3.3 Cumulative lifetime economic and health outcomes

*H. pylori*‐positive patients aged 60 had the highest cumulative lifetime economic and health outcomes (Table S1). From 2013 to 2019 for 8.50 million patients, *H. pylori* eradication saved US$3.75 billion, increased 11.11 million QALYs and 0.45 million LYs, and prevented 284,188 cases and 65,060 deaths. For 35.59 million patients without eradication, *H. pylori* eradication has the potential to save US$14.82 billion, increase 43.10 million QALYs and 1.66 million LYs, and prevent 1,084,532 cases and 250,256 deaths (Table S1).

In the Markov cohort analysis, the cumulative lifetime potential of gastric cancer cases and deaths from gastric cancer in *H. pylori eradication* strategy compared with no eradication strategy decreased by 30 to 33% in patients under 50 and by 25 to 28% in patients aged 50 and over (Figure S5). *H. pylori* eradication reduced the incidence and mortality of gastric cancer in the younger age groups greater than in the older age groups (Table 2, Figure S5).

## 4 DISCUSSION

To the best of our knowledge, this is the first study to assess the economic and health impacts of population‐wide *H. pylori* eradication strategy in national gastric cancer prevention program covered by National Health Insurance in the world.

We demonstrated that population‐wide *H. pylori* eradication strategy reduced costs, prevented gastric cancer, and reduced deaths from gastric cancer for all age groups in the modeling study with real‐life settings in Japan, even though most older adults with gastric mucosal atrophy require more than 10 years of follow‐up endoscopic screening after successful *H. pylori* eradication.^27^, ^28^ The cost savings of *H. pylori* eradication strategy from 2013 to 2019 were US$3.75 billion, 10.46 times the annual budget for cancer control in Japan. This means that the promotion of *H. pylori* eradication strategy focused on primary prevention of gastric cancer not only saves many lives from gastric cancer, but also leads to significant cost savings in the national budget.

It is well known that the benefits of *H. pylori* eradication on the reduction of gastric cancer risk in the younger age groups are greater than those in the older age groups. Young individuals would benefit most from *H. pylori* eradication because it cures *H. pylori* related gastritis, reduces the risk of gastric cancer, and reduces transmission to their children.^7^ This modeling study using the constant risk of gastric cancer development after successful eradication treatment demonstrated that *H. pylori* eradication reduced the incidence and mortality of gastric cancer in the younger age groups greater than in the older age groups. If we could modify to reduce the risk of gastric cancer development after successful eradication treatment in the younger age groups, more significant effects on reducing the incidence and mortality of gastric cancer could be shown in the younger age groups.

Surveillance of the local antibiotic resistance of *H. pylori* is recommended to identify the optimal empirical therapy for *H. pylori* eradication in the country.^7^ Chiang et al demonstrated no change of the antibiotic resistance rate of *H. pylori* through the selection of effective eradication regimens and retesting those who had completed *H. pylori* treatments in mass *H. pylori* eradication program.^29^ Guo et al found that successful *H*. *pylori* eradication potentially restored gastric microbiota to a similar status as found in uninfected individuals, and showed beneficial effects on gut microbiota.^30^ Liou et al showed that *H pylori* eradication had no effect on antibiotic resistance of *E coli* and no significant change in the prevalence of metabolic syndrome.^31^ These recent studies suggested that *H. pylori* eradication strategy with effective regimens and high compliance rates could provide significant benefits with minimal adverse effects in high‐risk countries.

Several economic analyses suggested that *H. pylori* screening followed by eradication treatment is cost‐effective to prevent gastric cancer, particularly in high‐risk populations.^32^, ^33^, ^34^, ^35^, ^36^, ^37^, ^38^, ^39^, ^40^ Han et al demonstrated that *H. pylori* screening and eradication treatment effectively reduced the morbidity of gastric cancer and cancer‐related costs in asymptomatic infected individuals in China.^33^ Chen et al showed that population‐based screen‐and‐treat strategy for *H pylori* infection proved cheaper and more effective for preventing gastric cancer, peptic ulcer disease, and nonulcer dyspepsia in asymptomatic general population compared with no‐screen strategy in China.^34^ Zheng et al found that *H. pylori* eradication treatment was an economical strategy with lower costs and greater efficacy in first‐degree relatives of patients with gastric cancer in China.^35^ Cheng et al demonstrated that *H. pylori* test‐and‐treat program was cost‐effective to prevent gastric cancer in an endemic area where *H. pylori* prevalence was >73.5% in Taiwan.^36^ Teng et al found that *H. pylori* screening was likely to be cost‐effective particularly for Māori in New Zealand.^37^ Beresniak et al showed that *H. pylori* test and eradication strategy including the use of urea breath test was the most cost‐effective compared to symptomatic treatment and upper gastrointestinal endoscopy in Spain.^38^ Our previous studies demonstrated the superior cost‐effectiveness of *H. pylori* screening with eradication, compared to no screening, upper gastrointestinal series, and endoscopic screening for asymptomatic general populations in Japan.^39^, ^40^

This study has several limitations. First, age‐specific numbers of patients with eradication were estimated based on database for Hokkaido (the north island of Japan), the expert opinion, and the literature.^10^, ^11^ Second, we did not consider reinfection and recurrence of *H. pylori* infection in the model. The reinfection rate after *H. pylori* eradication is very low. *H. pylori* infection is mainly transmitted in childhood, and recurrence of *H. pylori* infection after successful eradication is rare in adults.^41^ Third, nonmedical indirect costs, such as lost productivity, were not included in this study. Forth, we did not consider other risk factors of gastric cancer such as smoking, high salt intake, a diet low in fruit and vegetables, and genetic factors in this study. Fifth, the difference in the stage distribution of gastric cancer between different age groups was not included in the model. Sixth, we did not consider the histological changes after eradication in chronic gastritis patients in the model. *H. pylori* infection is well known to initiate sequential histological changes such as non‐atrophic gastritis, atrophic gastritis, intestinal metaplasia, dysplasia, and intestinal‐type gastric cancer. Diffuse‐type gastric cancer is also associated with *H. pylori* infection. Persistent inflammation results in the development of gastric atrophy. Earlier *H. pylori* eradication should be considered for preventing gastric cancer development prior to the appearance of precancerous lesions.^42^ *H. pylori* eradication strongly correlates with improvement in intestinal metaplasia in the antrum and gastric atrophy in the corpus and antrum of the stomach.^43^ More research is needed to incorporate the histological changes of gastric mucosa and future development of gastric cancer in chronic gastritis patients into the model. Finally, there are different costs, different epidemiological parameters, and medical systems in each country. Further cost‐effectiveness studies based on the variance of each country are required.

In conclusion, we demonstrated in the modeling study with real‐life settings that national policy using population‐wide *H. pylori* eradication to prevent gastric cancer has significant cost savings and health impacts for young‐, middle‐, and old‐aged individuals in Japan. The findings strongly support the promotion of *H. pylori* eradication strategy for all age groups in high‐incidence countries. Based on cost‐effectiveness, introducing *H. pylori* eradication strategy into national gastric cancer policy should be considered in high‐risk countries around the world.

## CONFLICTS OF INTEREST

The author has no conflicts of interest to declare.

## AUTHOR CONTRIBUTIONS

AK had full access to all the data in the study and takes responsibility for the integrity of the data and the accuracy of the data analysis. AK and MA approved the final version of the manuscript, and involved in concept, design, and critical revision of the manuscript for important intellectual content. AK involved in acquisition, analysis, interpretation of data, drafting of the manuscript, and administrative, technical, or material support. MA involved in supervision.

## Supporting information

Supplementary Material
