# Supplementary material for: Economic and health impacts of introducing Helicobacter pylori eradication strategy into national gastric cancer policy in Japan: A cost‐effectiveness analysis
Source: Helicobacter. 2021 Jul 18;26(5):e12837. doi: 10.1111/hel.12837 (PMC9286640; doi:10.1111/hel.12837)
Supplement: Supplementary file 1 — Supplementary Material [file HEL-26-0-s001.docx]

**Supplementary Table 1.** Cumulative lifetime economic and health outcomes.

| **Year** | **Age group**  **(y)** | ***H. pylori-*positive patients (n)** | **Cost-saving (US$)** | **QALYs gain (QALYs)** | **LYs gain (LYs)** | **Cases prevented gastric cancer (n)** | **Deaths averted gastric cancer (n)** |
| --- | --- | --- | --- | --- | --- | --- | --- |
| 2013-19 | 20 | 123,986 | 68,415,236 | 261,535 | 7,365 | 5,950 | 1,323 |
|  | 30 | 422,965 | 295,863,875 | 829,984 | 27,789 | 19,655 | 4,381 |
|  | 40 | 1,083,631 | 915,862,937 | 1,919,001 | 74,662 | 47,780 | 10,696 |
|  | 50 | 1,664,732 | 600,385,587 | 2,548,705 | 107,209 | 64,812 | 14,627 |
|  | 60 | 2,860,031 | 1,126,480,333 | 3,564,170 | 157,016 | 95,294 | 21,844 |
|  | 70 | 1,903,756 | 653,540,393 | 1,740,223 | 66,631 | 45,314 | 10,787 |
|  | 80 | 440,503 | 85,660,208 | 251,043 | 6,299 | 5,383 | 1,403 |
|  | Total | 8,499,603 | 3,746,208,570 | 11,114,661 | 446,971 | 284,188 | 65,060 |
| 2020- | 20 | 773,480 | 426,806,264 | 1,631,579 | 45,945 | 37,121 | 8,252 |
|  | 30 | 2,034,480 | 1,423,118,760 | 3,992,260 | 133,665 | 94,542 | 21,071 |
|  | 40 | 4,256,520 | 3,597,525,574 | 7,537,871 | 293,274 | 187,680 | 42,013 |
|  | 50 | 5,634,640 | 2,032,132,916 | 8,626,634 | 362,871 | 219,371 | 49,510 |
|  | 60 | 7,331,490 | 2,887,653,966 | 9,136,503 | 402,499 | 244,278 | 55,996 |
|  | 70 | 9,622,120 | 3,303,177,575 | 8,795,580 | 336,774 | 229,030 | 54,522 |
|  | 80 | 5,933,880 | 1,153,902,305 | 3,381,718 | 84,854 | 72,510 | 18,893 |
|  | Total | 35,586,610 | 14,824,317,360 | 43,102,145 | 1,659,883 | 1,084,532 | 250,256 |
| *H. pylori* = *Helicobacter pylori*; QALY = quality-adjusted life-year; LY = life expectancy life-year; | | | | | | | |

**(A)**


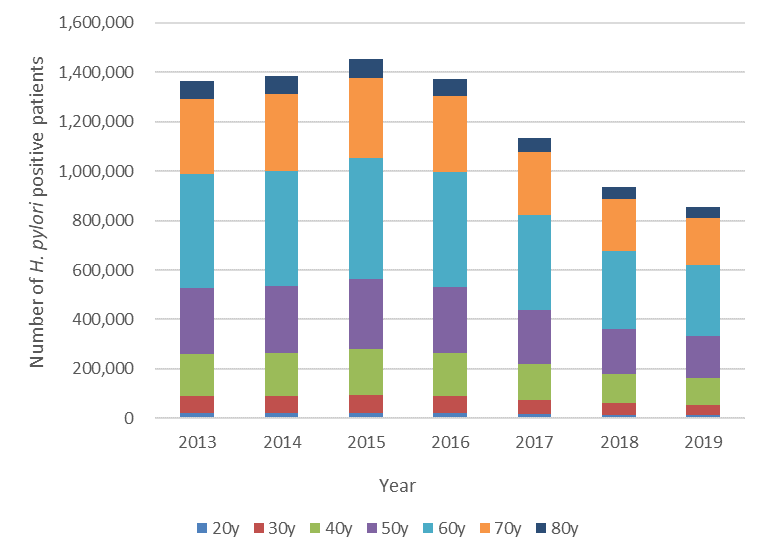


(B)


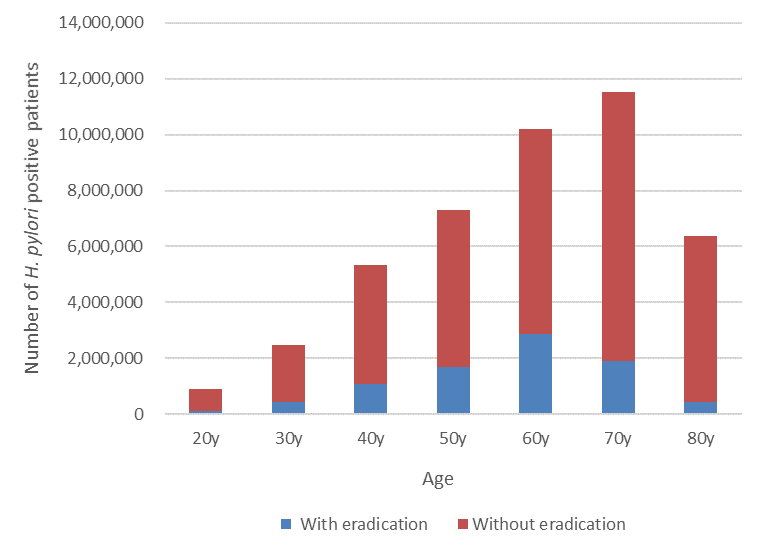


**Supplementary Figure 1.** A, Changes in the number of *H. pylori*-positive patients with eradication from 2013 to 2019. B, Age-specific number of *H. pylori*-positive patients with and without eradication. *H. pylori* = *Helicobacter pylori*;

**(A)**
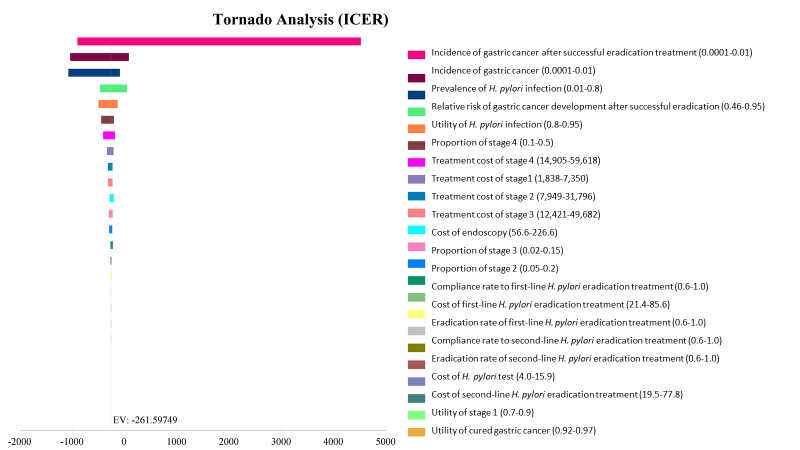


**(B)**
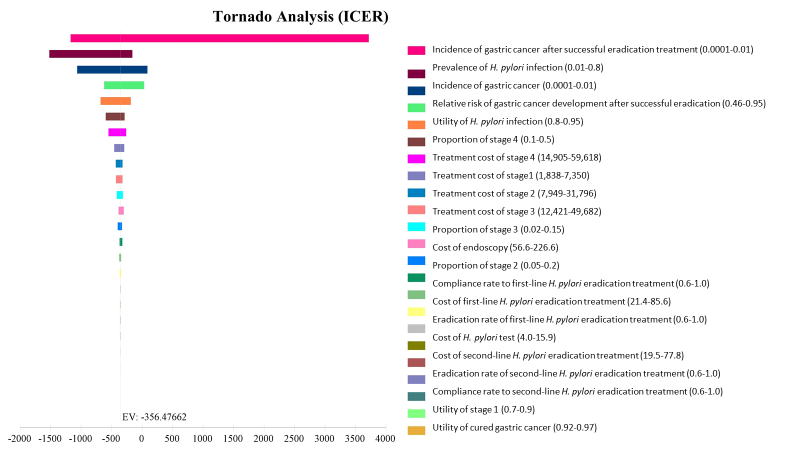


**(C)**
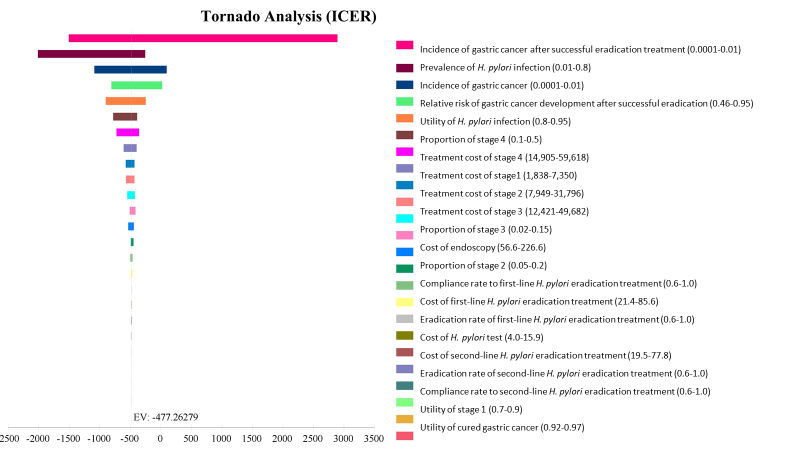


**(D)**
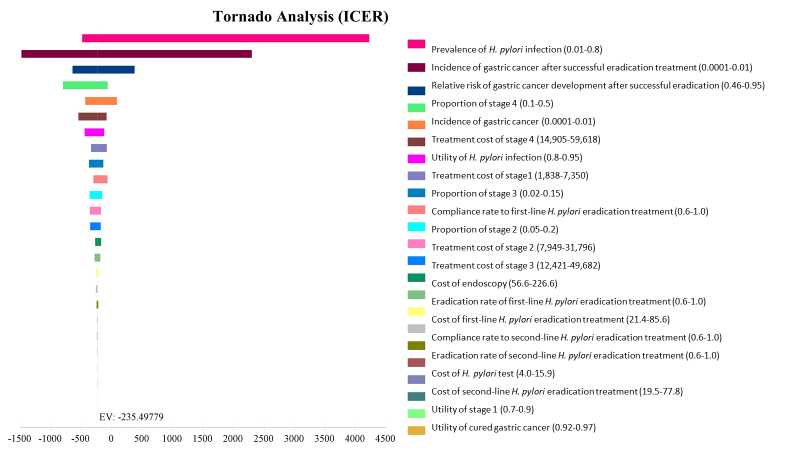


**(E)**
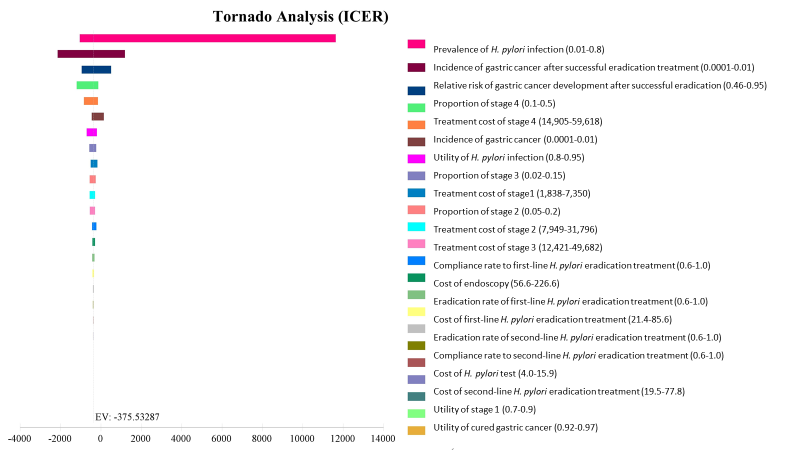


**(F)**
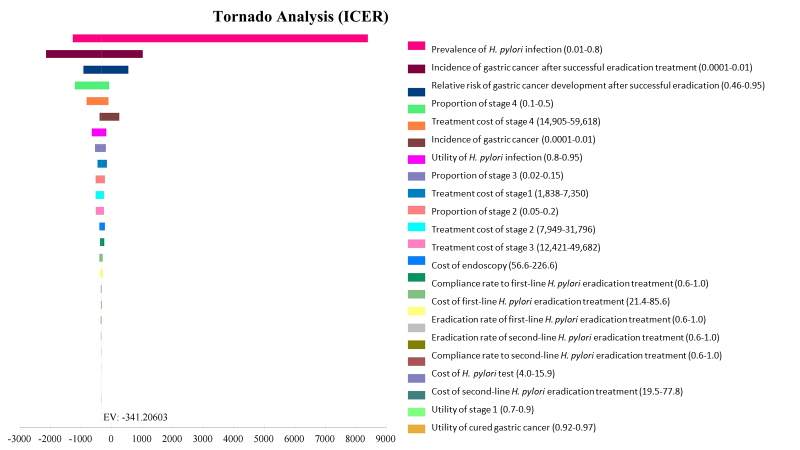


**Supplementary Figure 2.** The incremental cost-effectiveness ratio (ICER) tornado diagram for *H. pylori* eradication strategy versus no eradication strategy.

Incremental cost-effectiveness ratio tornado diagrams of *H. pylori* eradication strategy versus no eradication strategy showed that cost-effectiveness was not sensitive to any variables in all age groups. A, 20-year-old *H. pylori*-positive patients. B, 30-year-old *H. pylori*-positive patients. C, 40-year-old *H. pylori*-positive patients. D, 50-year-old *H. pylori*-positive patients. E, 70-year-old *H. pylori*-positive patients. F, 80-year-old *H. pylori*-positive patients. EV = expected value; *H. pylori* = *Helicobacter pylori*; ICER = incremental cost-effectiveness ratio;

**(A)**

**
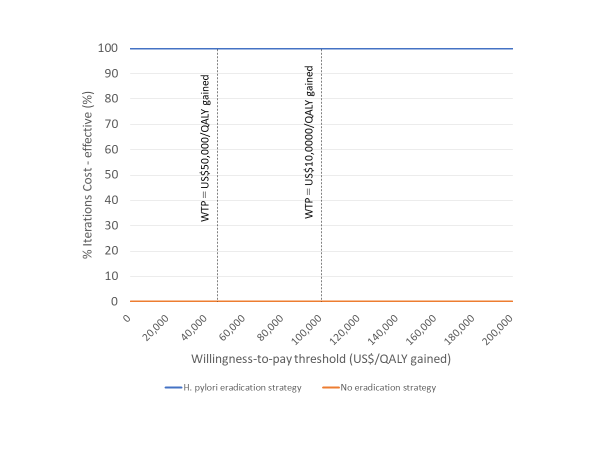
**

**(B)**

**
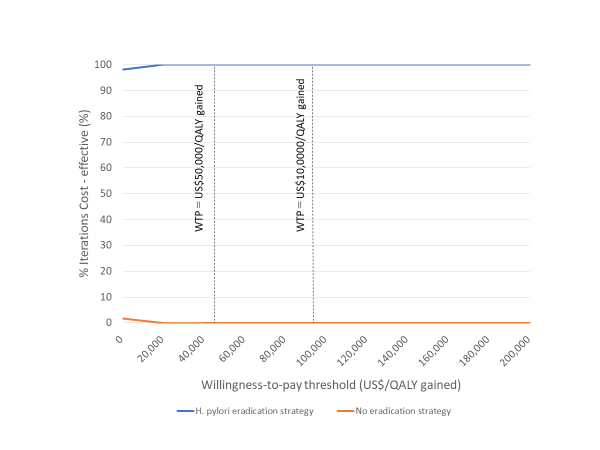
**

**Supplementary Figure 3.** Cost-effectiveness acceptability curve for *H. pylori* eradication strategy versus no eradication strategy.

The probabilistic sensitivity analysis analyzed 10,000 simulations of the model in which input parameters were randomly varied across pre-specified statistical distributions. The x-axis represents the willingness-to-pay threshold. The acceptability curves showed that *H. pylori* eradication strategy was cost-effective 100% of the time at two willingness-to-pay thresholds of US$50,000 per QALY gained and US$100,000 per QALY gained in all age groups. A, *H. pylori* positive-patients aged 20, 30, and 40. B, *H. pylori*-positive patients aged 50, 70, and 80. *H. pylori* = *Helicobacter pylori*; WTP = willingness-to-pay threshold;

**(A)**


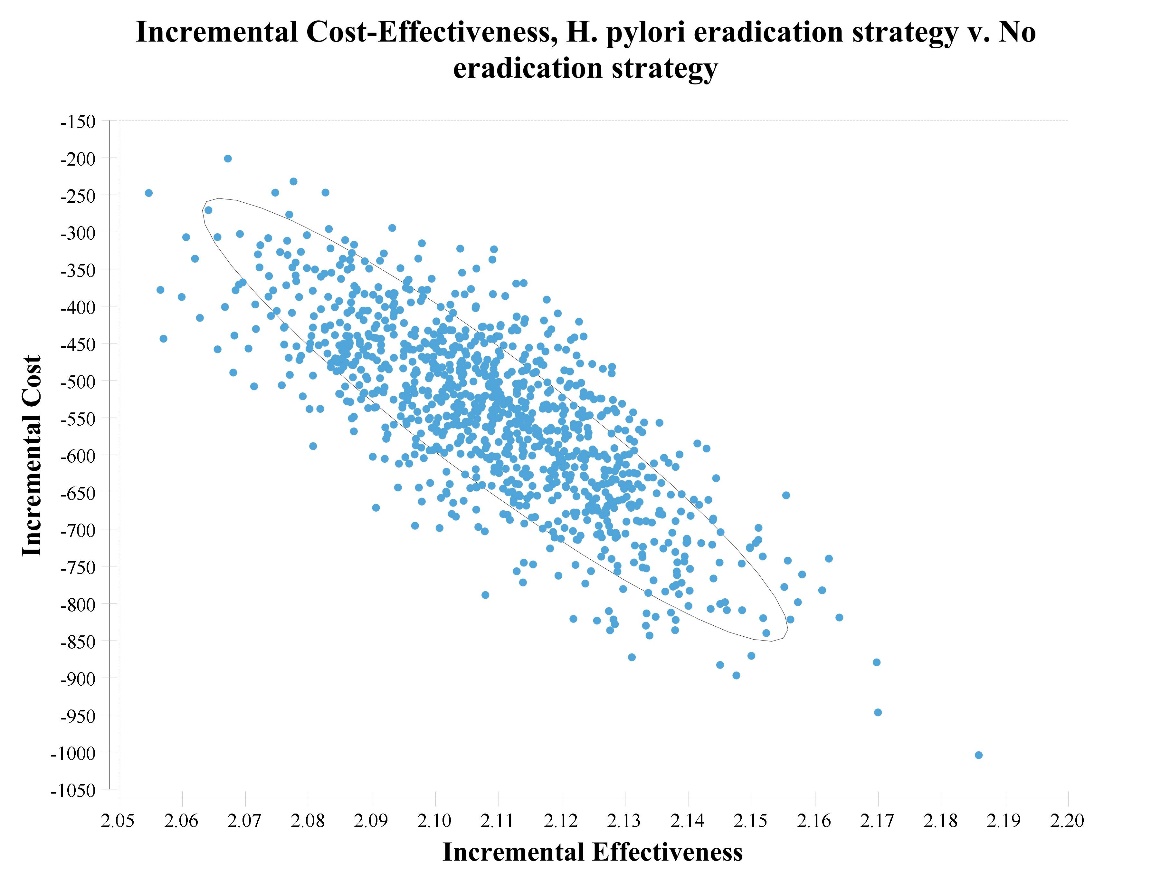


**(B)**


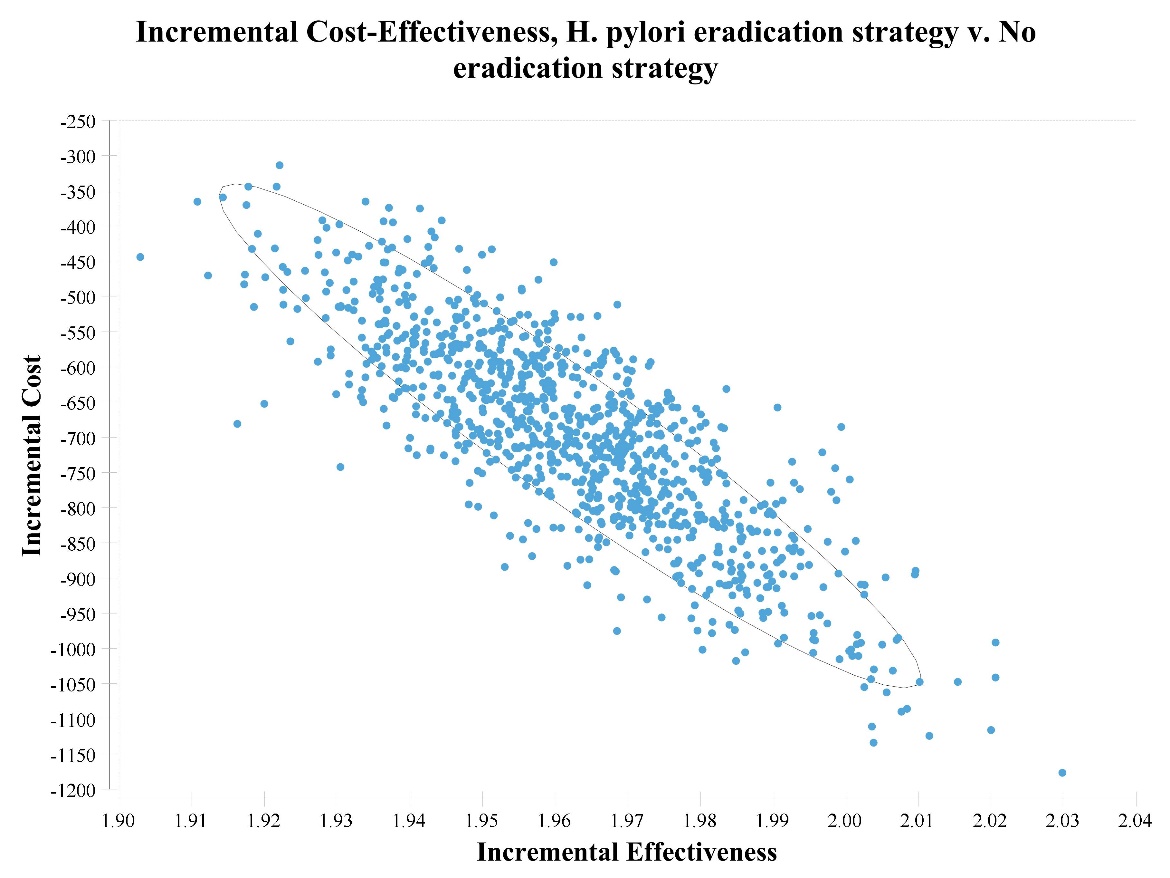


**(C)**


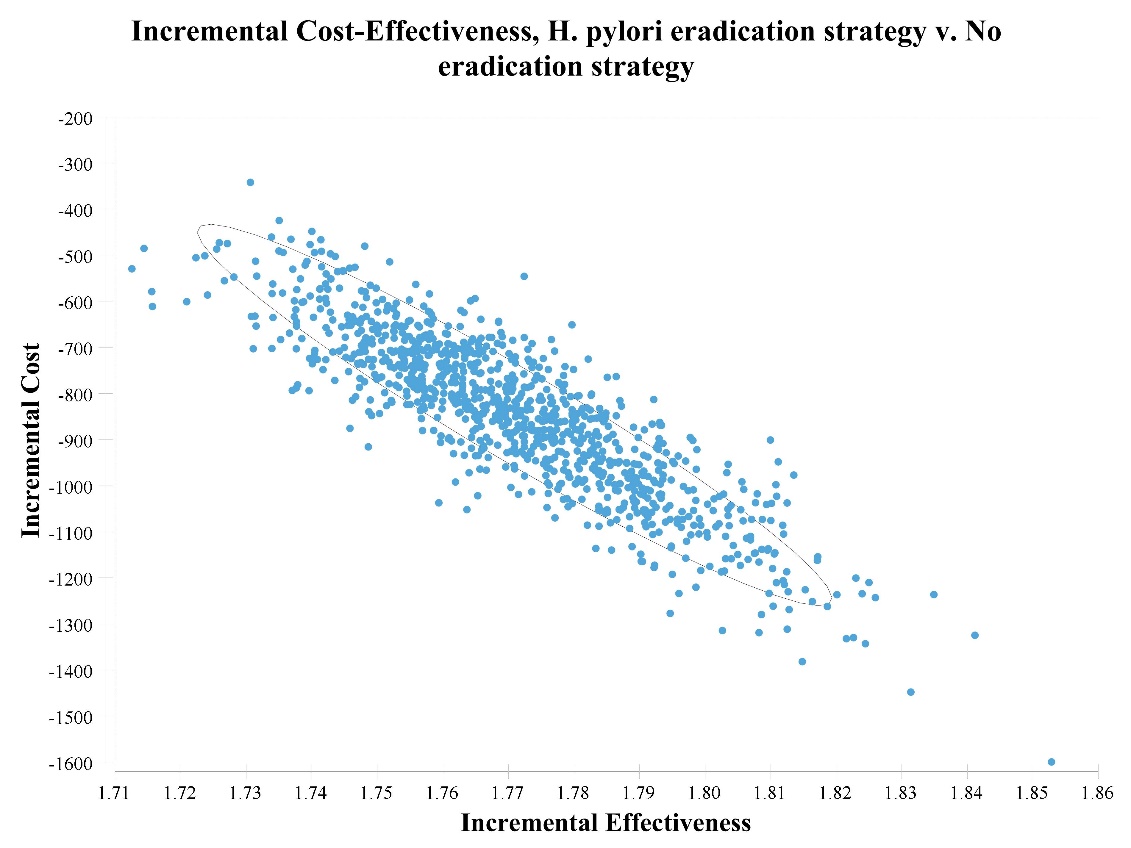


**(D)**


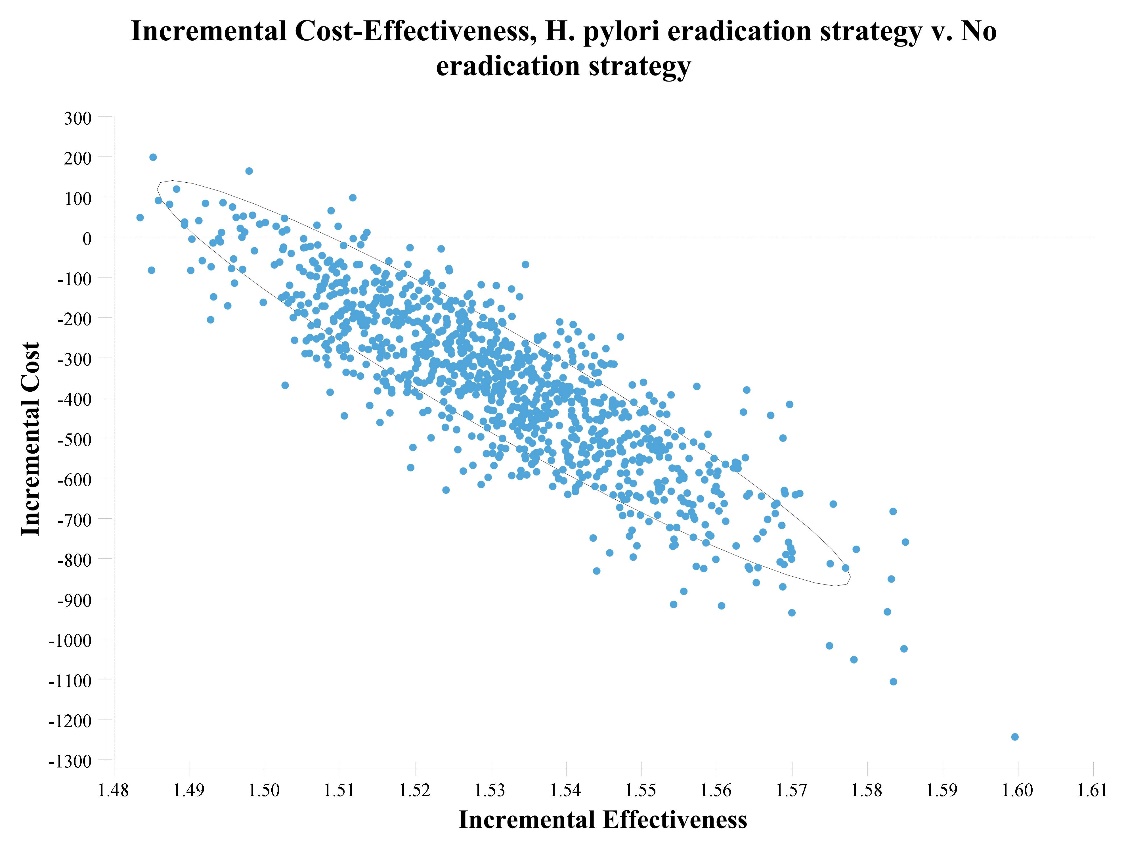


**(E)**


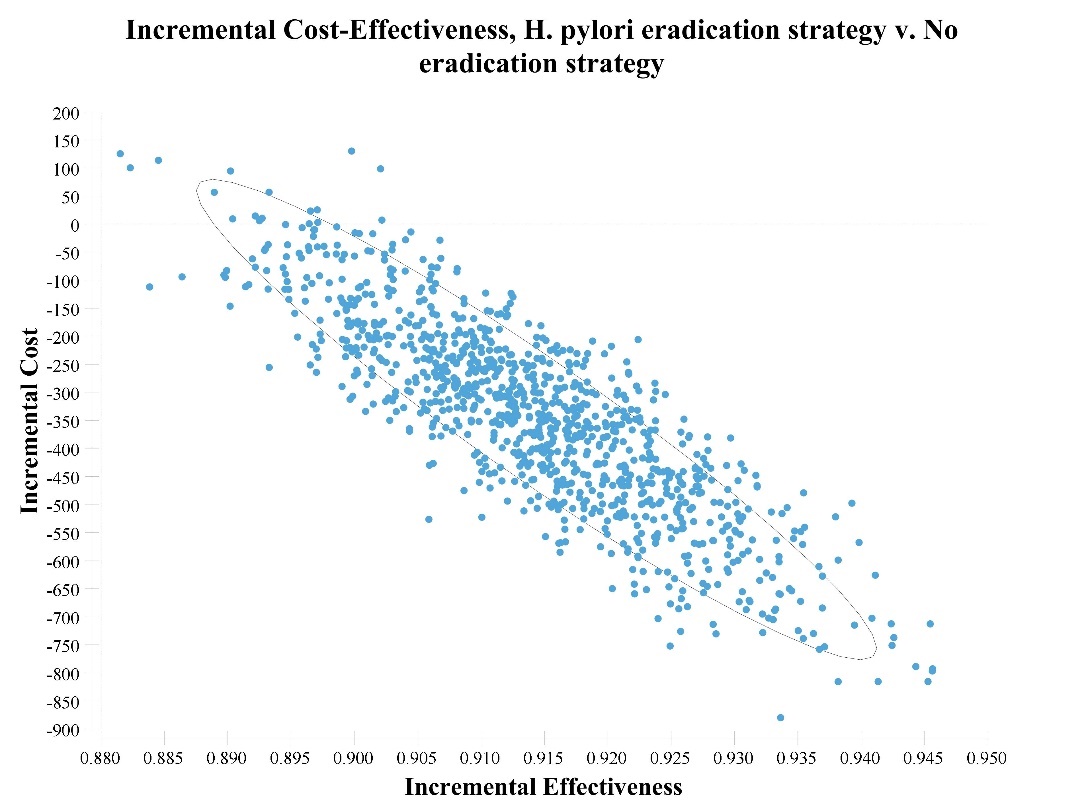


**(F)**


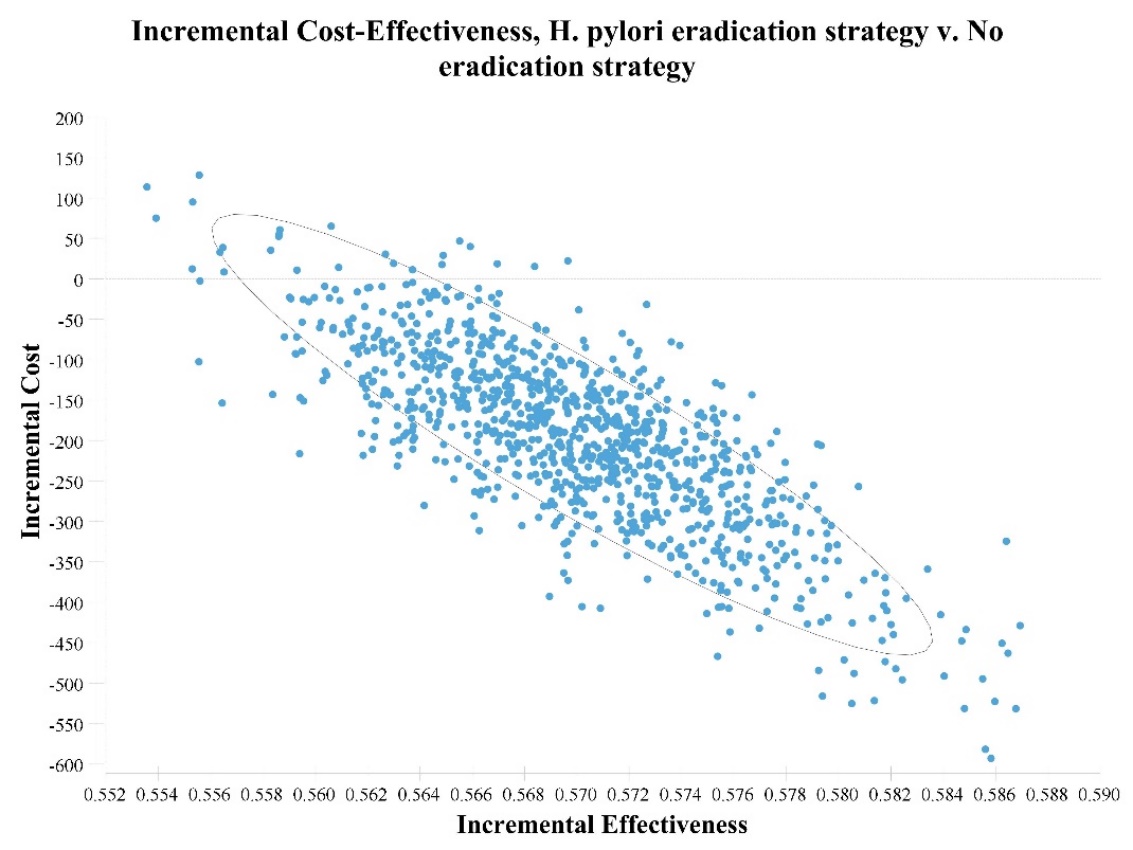


**Supplementary Figure 4.** Incremental cost-effectiveness scatterplots with 95% confidence ellipses for *H. pylori* eradication strategy versus no eradication strategy.

Each dot represents a single simulation for a total of 10,000 simulations. The x-axis represents incremental effectiveness and the unit is QALY. The y-axis represents incremental cost and the unit is US$. Incremental cost-effectiveness scatterplots showed that *H. pylori* eradication strategy dominated no eradication strategy in 10,000 trials in *H. pylori*-positive patients aged 20, 30, 40, and 50, and in more than 9800 trials in *H. pylori*-positive patients aged 50, 70, and 80. A, 20-year-old *H. pylori*-positive patients. B, 30-year-old *H. pylori*-positive patients. C, 40-year-old *H. pylori*-positive patients. D, 50-year-old *H. pylori*-positive patients. E, 70-year-old *H. pylori*-positive patients. F, 80-year-old *H. pylori*-positive patients. *H. pylori* = *Helicobacter pylori*; QALY = quality-adjusted life-year;


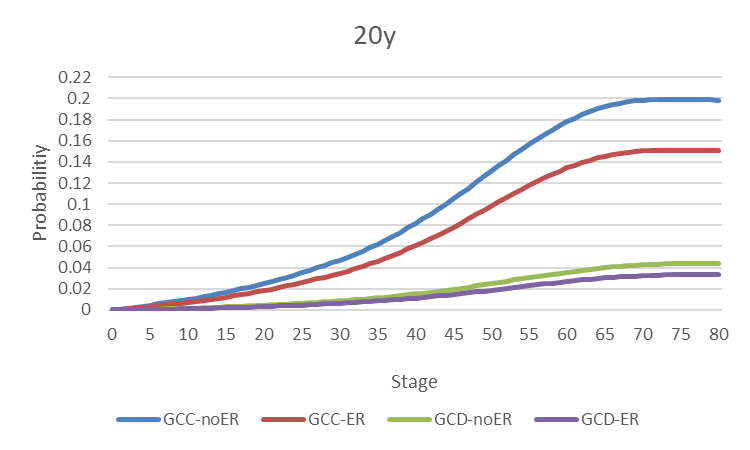


(B)


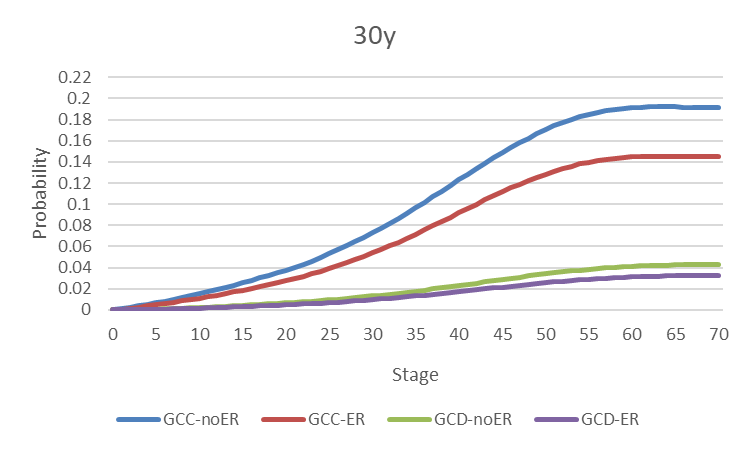


(C)


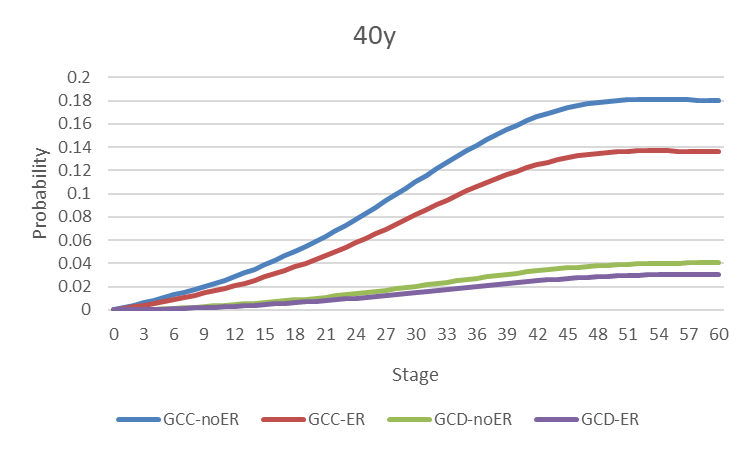


(D)


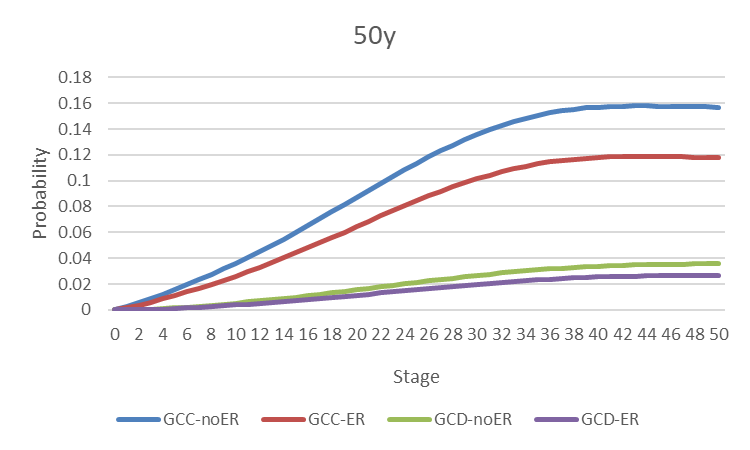


(E)


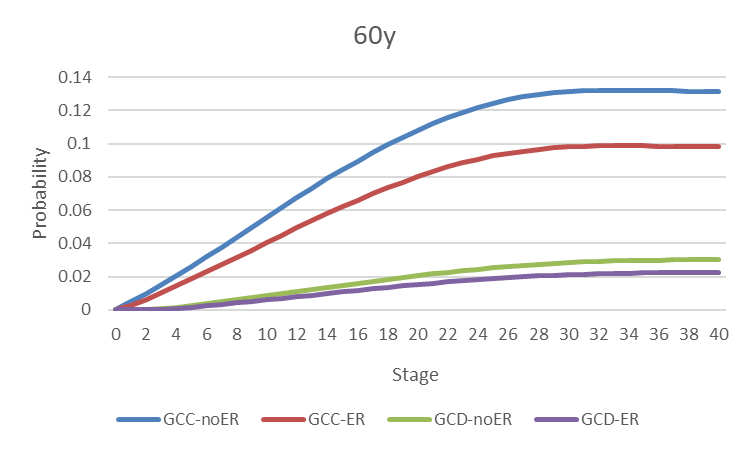


(F)


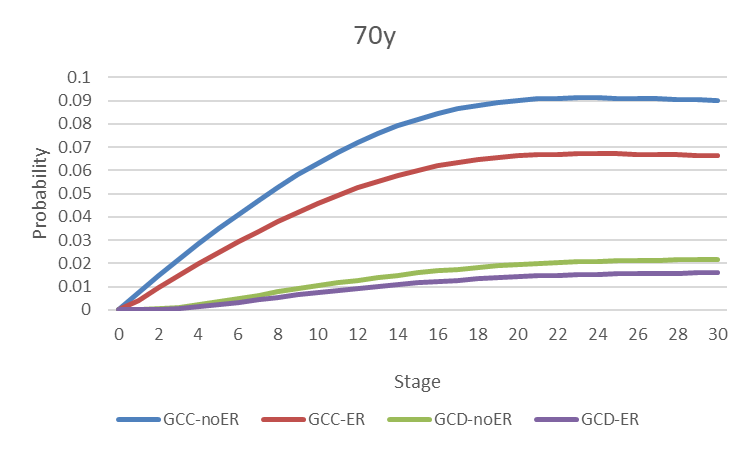


(G)


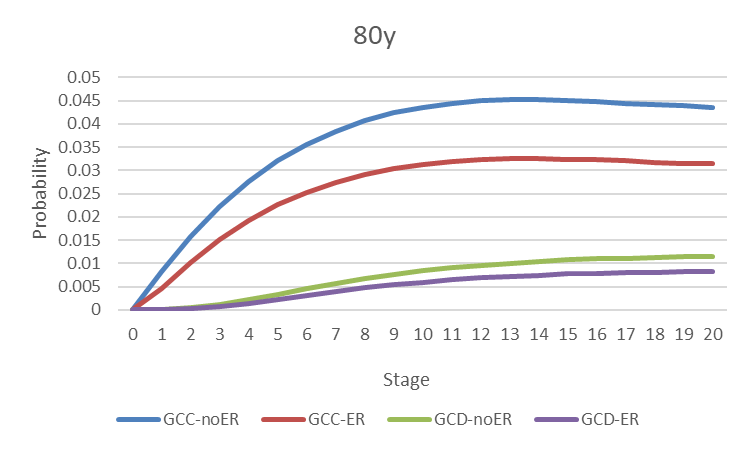


**Supplementary Figure 5.** Stage-specific changes of the cumulative potential of gastric cancer cases and deaths from gastric cancer in *H. pylori* eradication strategy and no eradication strategy in Markov cohort analysis. A, 20-year-old *H. pylori*-positive patients. B, 30-year-old *H. pylori*-positive patients. C, 40-year-old *H. pylori*-positive patients. D, 50-year-old *H. pylori*-positive patients. E, 60-year-old *H. pylori*-positive patients. F, 70-year-old *H. pylori*-positive patients. G, 80-year-old *H. pylori*-positive patients. GCC-noER = gastric cancer cases in no eradication strategy; GCC-ER = gastric cancer cases in *H. pylori* eradication strategy; GCD-noER = deaths from gastric cancer in no eradication strategy; GCD-ER = deaths from gastric cancer in *H. pylori* eradication strategy; *H. pylori* = *Helicobacter pylori*;
